# L-glutamine- and enzyme-supplementation via liquid feed to suckling piglets does not impact growth, health or intestinal structure

**DOI:** 10.1093/tas/txaf066

**Published:** 2025-05-16

**Authors:** Elisa A Arnaud, Gillian E Gardiner, John V O’ Doherty, Torres Sweeney, Peadar G Lawlor

**Affiliations:** Pig Development Department, Teagasc, Animal and Grassland Research and Innovation Centre, Moorepark, Fermoy, Co. Cork P61 C996, Ireland; Eco-Innovation Research Centre, Department of Science, South East Technological University, Waterford City, Co. Waterford X91 KOEK, Ireland; Eco-Innovation Research Centre, Department of Science, South East Technological University, Waterford City, Co. Waterford X91 KOEK, Ireland; School of Agriculture and Food Science, University College Dublin, Belfield, Dublin 4, D04V1W8, Ireland; School of Veterinary Medicine, University College Dublin, Belfield, Dublin 4, D04V1W8, Ireland; Pig Development Department, Teagasc, Animal and Grassland Research and Innovation Centre, Moorepark, Fermoy, Co. Cork P61 C996, Ireland

**Keywords:** antibiotics, feed additive, large litters, swine, weaning

## Abstract

The provision of liquid creep feed to suckling pigs has been shown to increase dry matter intake compared to dry creep feeding. The increased feed intake associated with liquid feeding makes it attractive as a means of delivering feed additives to suckling pigs to optimize growth and health. The objective of this study was to determine the effect of L-glutamine and enzyme supplementation of liquid creep feed on pig growth up to target slaughter weight (~120 kg), health and intestinal structure. Sixty sows and their litters were blocked on sow parity, previous number of piglets weaned and sow weight at day 107 of gestation, and the litters were randomly assigned to one of 3 dietary treatments: 1) liquid starter diet (control); 2) control diet supplemented with 10 g of L-glutamine per kg of starter diet (glutamine); and 3) control diet supplemented with a cocktail of enzymes (lipase, protease and α-amylase included at 160 Lipase units, 30,000 New Feed Protein units and 67.5 Kilo Novozymes units, respectively per kg of starter diet). Dietary treatments were fed from day 8 of age to weaning at day 28. Pig weight and dry matter disappearance (DMd) were recorded during lactation and post-weaning until pigs reached target slaughter weight (~120 kg) at 158 d of age. Carcass weight and quality were recorded. Medication usage, and the number of injections and clinical cases of disease were recorded from birth to slaughter. At day 5 post-weaning, a subset of pigs (n = 30) were sacrificed and intestinal samples were collected for histological analysis. The DMd of creep feed did not differ between treatments (*P* > 0.05). Glutamine tended to reduce piglet body weight (BW) at day 21 (*P* = 0.09) and 28 (*P* = 0.08) of lactation and from day 14 to 21, glutamine decreased piglet average daily gain (ADG) compared to the control (*P* < 0.05). Post-weaning growth was not affected by treatment (*P* > 0.05). The amount of antibiotics or anti-inflammatories administered to piglets or sows was not affected by treatment, either pre- or post-weaning (*P* > 0.05). However, glutamine tended to increase diarrhea prevalence between day 8 and 27 of lactation compared to the control (*P* = 0.09). In conclusion, supplementing liquid creep feed with glutamine tended to reduce pre-weaning growth and to increase diarrhea prevalence in piglets. Additionally, supplementing liquid creep feed with enzymes had no effect on growth or medication usage in pigs.

## Teaser

Providing a liquid diet supplemented with L-glutamine to suckling piglets tended to decrease their weaning weight but did not affect post-weaning pig growth. Providing a liquid diet supplemented with a cocktail of enzymes to suckling piglets did not benefit pre- or post-weaning pig growth.

## INTRODUCTION

Weaning is a challenging event for piglets. They are moved to an unfamiliar environment and the diet changes abruptly from sows’ milk, a highly digestible diet in liquid form, to a dry solid diet mainly of vegetable origin. This results in delayed and reduced feed intake and growth, and sometimes diarrhea in piglets during the early post-weaning period (Hampson and Smith, 1986; Wolter and Ellis, 2001; Collins et al., 2017). Providing dry supplementary feed (i.e., creep feed) to suckling piglets is a strategy to attempt to increase pre-weaning growth and weaning weight. Furthermore, it can familiarize suckling pigs with feed prior to weaning, thereby reducing latency to first feed after weaning and hence increasing post-weaning feed intake and growth (Pluske et al., 2007; Muns and Magowan, 2018). However, dry creep feed consumption varies greatly within and between litters and the response to creep feeding is particularly influenced by the proportion of “eaters” and “non-eaters” of creep-feed within the litter (Bruininx et al., 2002; Sulabo et al., 2010). Studies have shown that supplementing pigs with creep feed in liquid form has the potential to increase pre-weaning creep feed intake compared to dry creep feeding (Martins et al., 2020; Byrgesen et al., 2021; Vasa et al., 2023). The high feed intake with liquid creep feeding also means that it could be an efficient way to deliver non-antibiotic feed additives to suckling pigs to ensure optimal growth and health.

At weaning, transition from maternal milk to solid feed leads to a remodeling of the gastrointestinal tract. This includes a change in enzyme production; for example, for the intestinal brush-border disaccharidases this means a decrease in lactase activity and a concomitant increase in sucrase and maltase activity (Cunha, 1977). Enzymes with proteolytic activity are also found in low concentrations during the suckling period (Pierzynowski et al., 1990, 1993). Previous studies suggest that intestinal tract remodeling can be accelerated when an exogenous enzyme blend (amylase, protease and lipase) is fed to suckling mammals (Słupecka et al., 2012; Prykhodko et al., 2015, 2016). In addition, it was demonstrated that supplementing suckling pigs with a complex of microbially-derived amylase, protease and lipase can benefit pig growth and feed efficiency during the grow–finishing period (Prykhodko et al., 2016).

The reduced energy and nutrient intake experienced by pigs early post-weaning often leads to shortening of villus height and increased crypt depth in the small intestine (Miller et al., 1986). This reduces the surface area of the small intestine and the number of mature enterocytes, thereby limiting nutrient absorption (van Beers-Schreurs et al., 1992). Glutamine is a major fuel for enterocytes in the small intestine and can be metabolized into purines and pyrimidines for the synthesis of nucleotides to support cell proliferation (Watford, 2015). It was previously demonstrated that inclusion of L-glutamine at 1% (i.e., 10 g/kg) in dry creep feed can help to maintain intestinal structure and improve post-weaning feed efficiency (Cabrera et al., 2013). It has also been shown that inclusion of 1% L-glutamine in the diet of weaned pigs decreased diarrhea prevalence (Teixeira et al., 2014).

Therefore, supplementing suckling pigs with L-glutamine or a cocktail of enzymes (lipase, α-amylase and protease) could help to maintain intestinal structure and to accelerate intestinal tract remodeling at weaning. The objective of this study was to determine the effect of feeding liquid starter diets with or without supplementation with L-glutamine or a cocktail of enzymes to suckling pigs from day 8 of age on piglet growth, diarrhea prevalence and medicinal usage to weaning. Furthermore, the effect of dietary treatment on post-weaning growth, intestinal structure, diarrhea prevalence and medicinal usage to target slaughter weight was also determined. The hypothesis was that feeding liquid starter diets supplemented with L-glutamine or a cocktail of enzymes to suckling pigs from day 8 of age would increase pre-weaning growth and health and consequently reduce the need for injectable therapeutic (anti-inflammatory and antibiotic) use in piglets and improve intestinal structure post-weaning. Furthermore, it was hypothesized that these benefits would increase growth and carcass weight in pigs.

## MATERIAL AND METHODS

### Ethics Approval

This study was performed between June 2023 and January 2024, at the Teagasc Pig Development Department, Moorepark, Fermoy, Co. Cork, Ireland. Ethical approval for this study was granted by the Teagasc Animal Ethics Committee (approval no. TAEC2020-274) and South East Technological University Ethics Committee (approval no. WIT2021REC011). The project was authorized by the Irish Health Products Regulatory Authority (project authorization no. AE19132/P129). The experiment was conducted in accordance with the legislation for commercial pig production set out in the European Communities (Welfare of Farmed Animals) Regulations 2010 and in Irish legislation (SI no. 311/2010).

### Experimental Design and Animal Housing

Sixty sows (Large White × Landrace; PIC®, Hermitage Genetics, Sion Road, Co. Kilkenny, Ireland) were used in this study, which was conducted over three batches (with a batch being a group of sows inseminated on the same week). Sows were artificially inseminated at onset of standing estrus and again 24 hours later using pooled semen (Topigs Norsvin Tempo, Premier Pig Genetics Limited, Ireland). Gestating sows were housed in dynamic groups of ~120 animals. Sows were introduced to the dynamic group between 3 and 6 d after service and fed from electronic sow feeders [Schauer Feeding System (Competent 6), Prambachkirchen, Austria]. On day 107 of gestation, sows were blocked within farrowing batch into 20 pre-weaning blocks on the basis of parity group (mean ± SD; 2.5 ± 0.91), number of pigs weaned/sow in the previous cycle (13.7 ± 1.58 for multiparous sows) and body weight (BW) (283 ± 31.7 kg). Sow parity group distribution was as follows: group one, parity 0 (25%); group two, parity 1 to 2 (7%); group three, parity 3 to 5 (65%); and group four, parity 6 to 8 (3%). Within pre-weaning block, suckling litters were randomly assigned to the following pre-weaning dietary treatments: 1) starter diet provided as a liquid creep feed from day 8 of age to weaning (control), 2) control supplemented with 10 g of L-glutamine per kg (glutamine) and 3) control supplemented with a cocktail of enzymes (lipase, protease and α-amylase) (enzymes).

Prior to housing of the sows in the farrowing accommodation, each farrowing room was cleaned, disinfected, and allowed sufficient time to dry according to standard practices in the facility. At ~ day 108 of gestation, sows were moved into standard farrowing crates in pens (2.5 m x 1.8 m) with cast-iron slats under the sow and plastic slats with a water-heated floor pad for the piglets (BigDutchman; Vechta, Germany). Farrowing room temperature was maintained at 24 ± 3.0 °C at the time of farrowing and gradually reduced to 21 °C by day 7 of lactation. The temperature of the heat pads was 38 to 40 °C for the first 2 d after farrowing and was reduced by ~1 °C each day to 30 °C at 10 d after farrowing and it was maintained at this temperature until weaning. Artificial lighting was provided daily from 0800 hours to 1630 hours. The average number of piglets born alive was 15.7 ± 3.40 piglets. Where possible, litter size was standardized between 24 hours and 48 hours after parturition. The final number of piglets remaining on each sow at 48 hours postpartum was affected by the rearing capacity of each sow (i.e., the number of available functional teats) and the availability of foster sows to take surplus piglets. Piglets’ teeth were clipped within 24 hours of birth. On day 5 postpartum, tails were docked and all piglets were injected with 1 mL of iron (Gleptosil, Ceva Santé Animale, Libourne, France). Male pigs remained fully intact and piglets were weaned at day 28 ± 1.0 of lactation.

To study the residual effect of the pre-weaning dietary treatment in progeny, a subsample of 360 healthy pigs of > 5.0 kg BW (8.5 ± 1.40 kg) was selected at weaning. Within litter treatment groups, pens of 10 pigs of the same sex (entire male or female) of even weight were formed and blocked post-weaning by sex and BW. Pen groups for control (n = 12), glutamine (n = 12) and enzymes (n = 12) were moved to weaner accommodation at weaning. Pig BW, feed disappearance and health were monitored up to target slaughter weight (~120 kg). Weaner pens were equipped with fully slatted plastic floors (2.5 × 2 m) with automatic environmental control. Each pen had a shelf-type single-space (33 cm) wet-dry feeder (BA19100, Verba, Verbakel, The Netherlands) with inset nipple drinker and a supplementary bowl drinker (SS Drinker, Rotecna, Lleida, Spain). A spiked rubber ball (Easyfix Luna 142, Easyfix, Galway, Ireland) was provided for each pen as environmental enrichment. Temperature in the weaner rooms was maintained at 28 °C during the first week after weaning and reduced by 2 °C each week to 22 °C at the end of 4 wk. Ventilation was from a punched ceiling with air exhausted via a variable speed fan linked to a thermostat which was controlled by computer (Big Dutchman 135). At day 43 post-weaning, pen groups were moved to finisher accommodation. Finisher pens had fully slatted concrete floors (2.4 × 4.2 m) with automatic environmental control. Each pen had one shelf-type single-space (33 cm) wet-dry feeder (MA19100, Verba) with inset nipple drinker and a supplementary bowl drinker (SS Drinker, Rotecna). A wooden (larch) post was provided for each pen as environmental enrichment. All rooms were equipped with windows for natural light. Temperature in the finisher rooms was maintained at 20 to 22 °C with the same type of ventilation system used as in the weaner house. Pigs in each treatment group were slaughtered over 2 wk when they reached the target slaughter weight of ~120 kg live weight (LW; average age at slaughter 158 ± 3.2 d). The heaviest pigs in each pen group were slaughtered during the first week and the remaining pigs in the pen were slaughtered 7 d later.

### Diet Preparation and Feeding

Diets were formulated to meet or exceed National Research Council (NRC, 2012) recommendations. Diet samples were analyzed for dry matter (DM) (oven drying), ash (furnace drying and gravimetry), crude protein (Dumas method), total fat (Weibul acid hydrolysis) and crude fiber (Ankom 200 fiber analyzer, Macedon, New York, United States) by Sciantec Analytical Services Ltd, Selby, United Kingdom according to European Union Commission Regulation No 152/2009 (European Commission, 2009). The ingredient composition and chemical composition of the diets are shown in Table 1. During gestation, sows were fed a gestation diet (Diet 1, Table 1) in meal form at a feed allowance of 2.2 kg/day between day 0 and 90 of gestation. From day 90 of gestation to parturition, gestation feed allowance was increased to 2.7 kg/day. In the farrowing room, sows were fed a lactation diet (Diet 2; Table 1) in meal form using a computerized feed delivery system (DryExact Pro, Big Dutchman). Sows were fed twice daily from farrowing to day 6 of lactation and three times daily from day 7 to weaning at 28 d. Sows were fed according to a lactation feeding curve which started at 60 MJ digestible energy (DE)/d at day 0 of lactation and gradually increased to 107, 125, 133, and 137 MJ DE/d at days 7, 14, 21, and 26 of lactation, respectively. During lactation, feed allocation for individual sows was adjusted up and down from the curve, as necessary, to ensure that sow feed intake was as close as possible to ad libitum feed allowance and to prevent feed wastage. Between weaning and service, sows were provided with ad libitum access to the lactation diet (Diet 2, Table 1) for 4 d followed by the gestation diet (Diet 1; Table 1) in meal form. Water was provided on an ad libitum basis to sows from a single-bite drinker in the feed trough and to suckling piglets from a bowl (Big Dutchman) in the farrowing pen.

**Table 1.** Composition of the experimental diet (on an air-dry basis; kg/tonne)

Diet Number Diet type	1 Dry sow	2 Lactation	3^1^ Starter	4 Link	5 Weaner	6 Finisher
**Ingredients**						
Barley	747.5	288.6	50.0	77.0	292.7	589.7
Wheat	0	450.0	0	100.0	450	230.0
Maize	0	0	247	300	0	0
Soybean meal	74.4	173.8	141.1	180.7	150.2	146.1
Full fat soybean meal	0	0	125.0	75.0	50.0	0
Whey permeate	0	0	200	150	0	0
Skim milk powder	0	0	125	50	0	0
Soya hulls	141.9	0	0	0	0	0
Soya oil	10	49.2	77.5	31.0	23.5	10
Premix	1.5^2^	1.5^2^	3.0^3^	3.0^3^	3.0^3^	1.0^4^
L-Lysine HCl	2.18	5.1	6.4	6.8	6.3	4.7
DL-Methionine	0.5	1.6	3.6	3.2	2.1	1.3
L-Threonine	0.9	2.7	3.7	3.3	2.7	2.0
L-Tryptophan	0	0.8	1.4	1.3	0.6	0.3
L-Valine	0	2.6	1.3	1.2	0.7	0
Limestone flour	10	11.1	7.0	7.5	10.0	11.0
Mono dicalcium phosphate	7.2	8.0	5.0	7.0	5.3	1.0
Salt	4	5	3	3	3	3
Phytase^5^	0.1	0.1	0.1	0.1	0.1	0.1
**Chemical composition**						
Dry matter^6^	883.0	882.0	925.0	904.0	890.5	880.0
Crude protein^6^	117.0	168.0	191.0	174.0	162.5	163.0
Ash^6^	47.0	42.0	57.0	51.0	44.0	46.5
Ether extract^6^	34.8	69.9	115.7	83.4	74.8	36.6
Crude fibre^6^	83.0	30.0	18.0	19.5	31.0	33.5
Lysine^7^	7.8	11.5	16.2	15.0	13.0	10.9
Methionine^7^	2.4	3.9	7.0	6.1	4.7	3.4
Cystine^7^	2.5	3.0	2.7	2.9	3.1	3.1
Threonine^7^	5.6	8.3	10.9	10.1	8.8	7.6
Tryptophan^7^	3.66	3.36	2.66	2.22	1.54	2.79
Digestible energy (MJ/Kg)^7^	13.20	15.20	16.20	15.00	14.27	13.73
Net energy (MJ/Kg)^7^	8.59	10.93	12.06	10.94	10.30	9.80
SID lysine^7^^,^^8^	6.6	10.7	15.3	14.1	12.0	10.0
Total calcium^7^	7.2	8.3	8.2	7.5	7.4	6.5
Digestible phosphorus^7^	3.5	3.8	4.6	4.2	3.3	2.5

^1^The starter diet was used to prepare the liquid creep feed supplemented to piglets pre-weaning. In the liquid creep feed supplemented with glutamine, L-glutamine (Ajinomoto, Tokyo, Japan) was included in the starter diet at 10 g/kg of diet. In the liquid creep feed supplemented with the enzyme cocktail, the enzyme cocktail containing lipase (Capalase Micro R800, DSM-Firmenich, Heerlen, The Netherlands), protease (ProAct 360, DSM-Firmenich) and α-amylase (RONOZYME HiStarch, DSM-Firmenich) was included in the starter diet at 0.725 g/kg of diet so that the finished diet contained 160 lipase units (PLI)/kg, 30,000 new feed protease units (NFP)/kg and 67.5 Kilo Novo (α-amylase) units (KNU)/kg, respectively.

^2^Premix provided per kilogram of complete diet (Diets 1 and 2): Cu from copper sulphate, 15 mg; Fe from ferrous sulphate monohydrate, 70 mg; Mn from manganese oxide, 62 mg; Zn from zinc oxide, 80 mg, I from calcium iodate, 0.6 mg; Se from sodium selenite, 0.2 mg; vitamin A as retinyl acetate, 3.44 mg; vitamin D3 as cholecalciferol, 25 mg; vitamin E as DL-alpha-tocopheryl acetate, 100 mg; vitamin K, 2 mg; vitamin B12, 15 μg; riboflavin, 5 mg; nicotinic acid, 12 mg; pantothenic acid, 10 mg; choline chloride, 500 mg; biotin, 200μg; folic acid, 5mg; vitamin B1,2 mg; and vitamin B6, 3 mg.

^3^Premix provided per kilogram of complete diet (Diets 3, 4 and 5): Cu from copper sulphate, 100 mg; Fe from ferrous sulphate monohydrate, 90 mg; Mn from manganese oxide, 47 mg; Zn from zinc oxide, 120 mg; I from potassium iodate, 0.6 mg; Se from sodium selenite, 0.3 mg; vitamin A as retinyl acetate, 2.1 mg; vitamin D3 as cholecalciferol, 25 μg; vitamin E as DL-alpha-tocopheryl acetate, 100 mg; vitamin K, 4 mg; vitamin B12, 15 μg; riboflavin, 2 mg; nicotinic acid, 12 mg; pantothenic acid, 10 mg; choline chloride, 250 mg; vitamin B1, 2 mg; and vitamin B6, 3 mg.

^4^Premix provided per kilogram of complete diet (Diet 6): Cu from copper sulphate, 15 mg; Fe from ferrous sulphate monohydrate, 24 mg; Mn from manganese oxide, 31 mg; Zn from zinc oxide, 80 mg; I from potassium iodate, 0.3 mg; Se from sodium selenite, 0.2 mg; vitamin A as retinyl acetate, 0.7 mg; vitamin D3 as cholecalciferol, 12.5 µg; vitamin E as DL-alpha-tocopheryl acetate, 40 mg; vitamin K, 4 mg; vitamin B12, 15 µg; riboflavin, 2 mg; nicotinic acid, 12 mg; pantothenic acid, 10 mg; vitamin B1, 2 mg; vitamin B6, 3 mg.

^5^The diet contained 1000 phytase units (FYT) per kg feed (RONOZYME HiPhos GT DSM, Belfast, UK).

^6^Analysed nutrient composition.

^7^Calculated nutrient composition.

^8^ SID lysine = Standardized ileal digestible lysine.

Each of the starter diets (Diet 3, Table 1) was provided as a liquid creep feed to suckling piglets from day 8 to 28 of age. In the liquid creep feed supplemented with L-glutamine (Ajinomoto, Tokyo, Japan), it was included in the starter diet (Diet 3; Table 1) at 10 g/kg of diet, as previously described (Cabrera et al., 2013). In the liquid creep feed supplemented with the enzyme cocktail, the enzyme cocktail containing lipase (Capalase Micro R800, DSM-Firmenich, Heerlen, The Netherlands), protease (ProAct 360, DSM-Firmenich) and α-amylase (RONOZYME HiStarch, DSM-Firmenich) was included in the starter diet (Diet 3, Table 1) at 0.725 g/kg of diet so that the finished diet contained 160 lipase units (PLI)/kg, 30,000 new feed protease units (NFP)/kg and 67.5 Kilo Novo (α-amylase) units (KNU)/kg, respectively, according to the manufacturers' recommendations. Recovery tests for free glutamine, free glutamic acid, total glutamic acid, protease and α-amylase were conducted on the dry starter diet to confirm correct inclusion of these additives in the feed according to the International Organization for Standardization (ISO) NF EN ISO13903:2005 standard (International Organization for Standardization, 2005) (see Table S1). Additionally, recovery tests were conducted on liquid starter diet samples prepared by mixing dry pelleted starter diet with warm water at the same temperature and ratio as the ones used in experimental conditions and incubating for 5 hours at 30 °C. Before conducting these analyses, the liquid starter diet was oven dried at 55 °C for 3 consecutive days. The enzyme activities were determined using DSM-Firmenich proprietary procedures, with specific assay conditions (pH, temperature, buffers) adapted for the determination of DSM-Firmenich product activities.

The liquid starter diet provided as creep feed was prepared by mixing dry pelleted starter diet with warm water (55 °C) at a ratio of 1:5 and provided through an automatic feeding system (Babyfeed, Schauer Agrotronic GmbH, Prambachkirchen, Austria). The liquid feeding trough was positioned to one side of the sow’s head at the front of the farrowing pen. Fresh feed was prepared twice daily at 0835 hours and at 1645 hours. The starter diet was mixed with water at 55 °C for ~10 min. Ten feeding cycles (each lasting ~ 2 hours) were programmed between 0930 hours and 0400 hours. During each cycle the in-situ trough sensors checked the amount of liquid feed present in the trough five times. Whenever the feed level was below the level of the sensor, the trough was detected as empty, and the liquid starter diets were delivered to the trough and the amount delivered to the trough was recorded in the system computer at each re-fill. Therefore, each pen could potentially have been supplied with liquid starter diet up to 50 times in a 24-hour period. Each day after the last feeding, the system was cleaned in closed circuit, which included mixing tanks and all of the pipelines, with a 1% acid solution (Deosan Acidbrite AG313, Diversey Europe Operations BV, Utrecht, The Netherlands). In addition, the system was cleaned once a week with a 0.5% solution of an alkaline detergent (AvalKsan Gold Standard CF, Carbon Group, Ringaskiddy, Ireland), to help remove lime scale from the circuit. The troughs were cleaned each morning with air pressure and rinsed with acidified water and a 0.5% solution of the alkaline detergent was applied to troughs, as for the acid rinse, once weekly.

Following weaning, pigs were fed a sequence of diets in accordance with their growth stage. Starter diet (Diet 3; Table 1) was provided from weaning to day 6 post-weaning, link diet (Diet 4; Table 1) from day 6 to 17 post-weaning, weaner diet (Diet 5; Table 1) from day 17 to 43 post-weaning, and a finisher diet (Diet 6; Table 1) from day 43 post-weaning to slaughter (~ day 132 post-weaning). All diets for post-weaning pigs were provided in dry pelleted (3 mm diameter) form and access was on an ad libitum basis. Pigs were inspected daily and any pig demonstrating visual signs of illness was treated appropriately. Assessment of clinical signs of disease and treatment protocols were followed in accordance with farm protocol. All veterinary treatments were recorded, including antibiotic and anti-inflammatory treatments.

### Data Recording and Sampling

#### Sow body weight and back fat thickness.

Sow BW and back fat (BF) were recorded on day 108 of gestation, at weaning, and at their subsequent service (~day 4 post-weaning). Sow BW was recorded using an electronic sow scales (EziWeigh 7i, O’Donovan Engineering, Co. Cork, Ireland). Empty farrowing weight was calculated and body fat was measured as outlined by Arnaud et al. (2023).

#### Farrowing performance and pre-weaning piglet growth performance.

The individual weight and sex of each piglet was recorded at birth, when each piglet was tagged for identification purposes, on day 8, 14, 21 and 27 postpartum using an electronic piglet scale (Defender 3000 XtremeW, O’Donovan Engineering). These data were used to determine piglet pre-weaning average daily gain (ADG). The creep feed dry matter disappearance (DMd) was recorded daily. The net energy intake, lysine intake and L-glutamine intake from the creep feed was calculated based on DMd.

#### Live observation of trough-directed behavior per litter.

The feeding behavior of individual piglets within pen groups was observed in Batch 1 of the experiment using instantaneous scan sampling at day 13, 16 and 22 after birth. To enable the easy identification of piglets during scan sampling, piglets in each litter were marked with a number from 1 to 17 (linked to their tag number) using black hair dye (Pro Color Plus, Healthpoint, Blackpool, United Kingdom) on the day before scan sampling was conducted. Six 1-hour sessions were conducted between 0900 hours and 1600 hours on each scan sampling day. During each 1-hour session of live observations, each pen was scanned (i.e., the behavior of the group was recorded) every 3 min, leading to 21 scans/pen/session. A simple ethogram was used for scoring feeding behavior. At every scan, liquid trough-directed activity was recorded. Trough-directed activity was defined as when a piglet snout was immersed in the liquid feed for at least 2 s. Piglets were categorized as “eaters” using two different thresholds 1) if they were engaged in two or more trough-directed activities at any time during an observation day or 2) if they were engaged in one or more trough-directed activities at any time during an observation day. This is a proxy for classifying piglets eating creep feed or not. The percentage of piglet eaters per pen was calculated on a pen basis for each observation day and for all observation days combined. This was carried out by expressing the number of piglets considered as eaters in a pen as a percentage of the total number of piglets present in the pen.

#### Post-weaning pig growth performance and carcass data.

Pen groups were weighed on day 0 (weaning), 6, 14, 21, 28 and 43 post-weaning and individual pig weights were recorded just prior to slaughter (at ~day 132 post-weaning) using an electronic scale for weaners (Munster Weigh-Bridge Services Limited, Watergrasshill, Ireland) from day 0 to 43 post-weaning, and an electronic scale for finishers (GlobeWeigh, Cork, Ireland) from day 43 to 132 post-weaning with an electronic weight indicator (EziWeigh 7i, O’Donovan Engineering). Pigs were fasted for 18h prior to recording BW before slaughter. Feed disappearance was recorded on a pen basis between weaning and slaughter for the periods for which BWs were recorded. These data were used to determine the average daily feed intake (ADFI), ADG, and gain to feed ratio (G:F).

At day 132 post-weaning, pigs were transported 95 km to the abattoir (Dawn Pork & Bacon, Grannagh, Co. Waterford, Ireland) where they were killed by exsanguination after CO_2_ stunning. At the abattoir, carcass cold weight of individual pigs was calculated by multiplying the hot carcass weight, recorded within 45 min of the pig being exsanguinated, by 0.98. Muscle depth and BF measured at 6 cm from the edge of the split back at the level of the third and fourth last rib were determined using a Hennessy Grading Probe (Hennessy and Chong, Auckland, New Zealand). Lean meat content was calculated according to the following formula: Estimated lean meat content (%) = 60.3 − 0.847x + 0.147y, where x = fat depth (mm); y = muscle depth (mm) (Department of Agriculture and Food and Rural Development, 2001). Carcass ADG, carcass G:F and lean ADG were measured as outlined by Arnaud et al. (2023).

#### Faecal scoring and medication usage.

Faecal consistency scores on a pen basis were determined weekly at day 8, 14, 21 and 27 before weaning and on days 1, 5, 14, 21 and 28 post-weaning. A 4-point scoring system (Casey et al., 2007) was used and the average score from visual assessment of freshly voided feces on the pen floor from five pigs was determined as the average score for each litter/pen. In brief: 0 = normal (dry pelleted feces), 1 = soft (soft with shape), 2 = mild diarrhea (very soft or viscous liquid) and 3 = severe watery diarrhea (watery or with blood). The diarrhea prevalence at each time-point was determined by considering a fecal score of 2 or greater as indicative of diarrhea for each litter/pen.

Antibiotic and anti-inflammatory usage was recorded in sows during lactation and in pigs from day 8 of age until they reached their target slaughter weight [separately for the pre-weaning (day 8 to 28), weaner (day 0 to 43 post-weaning) and finisher (day 44 to 132 post-weaning) periods]. Medication was administered (when joint-ill, lameness, malaise or diarrhea were observed) by trained farm technicians in piglets and when malaise or vaginal discharge was observed in sows. One antibiotic (Unicillin; Univet Ltd, Cootehill, Co. Cavan, Ireland) and one anti-inflammatory (Loxicom, Norbrook, Newry, United Kingdom) only, were used during this experiment. Animal ID, pen number, product name, product code, dose administered (ml), frequency of administration, date of administration, and reason for use were recorded when an animal was treated. From this, the total number of piglet injections per litter/pen, the average amount of medication (antibiotic/anti-inflammatory) administered per pig on a litter basis and per sow, and the total number of clinical cases of disease (i.e., when an animal was treated one or more times for the same reason) per litter were calculated before weaning. The average amount of medication (antibiotic and anti-inflammatory) administered per pig per pen and the total number of clinical cases of disease were also calculated between weaning and target slaughter weight.

#### Euthanasia, blood and tissue sampling.

On day 5 post-weaning, 30 pigs (10 female piglets per treatment of even weight), were euthanized using captive bolt followed by immediate exsanguination. Blood was collected into Ethylene Diamine Tetra Acetic acid-coated 10 mL tubes (Becton Dickinson, Franklin Lakes, New Jersey, United States) and stored at room temperature for 7 to 9 hours until analysis. After euthanasia, the intestinal tract was removed and whole tissue samples (~2 cm) were collected from the duodenum (15 cm distal to the pyloric junction), jejunum (1.5 m distal to the pyloric junction) and ileum (15 cm proximal to the ileo-caecal junction). Each whole tissue sample was carefully immersed in a 50 mL tube containing NOTOXhisto™ fixative (Scientific Device Laboratory, Des Plaines, United States). The tubes were placed on a shaker at room temperature for 48 hours after collection and stored at room temperature until analysis.

### Laboratory Analyses

#### Haematology analyses.

Haematological analysis of the whole blood samples collected from euthanised piglets was performed using a Mythic 5 Vet Pro analyzer (Cormay diagnostics, Warsaw, Poland). The following parameters were measured: leukocyte number, lymphocyte number and percentage, monocyte number and percentage, neutrophil number and percentage, eosinophil number and percentage, basophil number and percentage, erythrocyte number, hemoglobin (g/dL), mean corpuscular volume (fL), mean corpuscular hemoglobin (pg/cell), mean corpuscular hemoglobin (g/dL) and platelet number.

#### Small intestinal histology.

The whole tissue samples from the duodenum, jejunum and ileum were sent to Nationwide Laboratories, Devon, UK for histological slide preparation with haemotoxylin-eosin staining to study gross morphological parameters of intestinal structure. For each sample, the villus height (VH) and crypt depth (CD) were measured, as described previously by Crespo-Piazuelo et al. (2021) and the ratio of VH to CD was calculated.

### Statistical Analysis

All data were tested for normality prior to analysis by examination of histograms and normal distribution plots using the univariate procedure. Residuals were inspected in all models to confirm normality. All data, except for the prevalence of diarrhea, were analyzed using the PROCMIXED procedure in the Statistical Analysis Systems (SAS) software package version 9.4 (SAS Institute Inc., Cary, North Carolina, United States). The prevalence of diarrhea per litter in the farrowing accommodation from day 8 to 28 and in the weaner accommodation per pen from weaning to day 28 post-weaning was analyzed using the PROC Glimmix procedure of SAS with a binomial distribution. Model fit was determined by choosing models with the minimum finite-sample corrected Akaike Information Criteria (AIC). The covariance structures chosen as having the lowest AIC are presented in Table S2. The only co-variates used in the same model were “litter size at day 8 pre-weaning” and “BW at day 8 pre-weaning” as shown in Table S2. The variance inflation factor (VIF) value for the co-variates “litter size at day 8 pre-weaning” and “BW at day 8 pre-weaning” was determined using the PROC REG procedure of SAS. The VIF for these co-variates was 1 indicating that there was no correlation between them.

For the analysis of pre-weaning litter weight, piglet BW, DMd per pig on a litter basis, sow BW and sow BF, the percentage of piglets classified as “eaters,” the total number of piglet injections per litter, the average amount of medication (antibiotic and anti-inflammatory) administered per pig on a litter basis and per sow, the total number of clinical cases of disease per litter or per pen, number of deaths and removals per litter, post-weaning growth parameters, carcass quality data and the average amount of medication (antibiotic and anti-inflammatory) administered per pig on a pen basis post-weaning and diarrhea prevalence; treatment was included in the model as a fixed effect.

For analysis of pre-weaning piglet growth parameters, piglet weight and litter size at day 8 were included as co-variates, when significant in the model. For analysis of sow BW and BF, initial value at day 108 of gestation was included as a covariate in the model.

For the analysis of post-weaning growth and carcass quality parameters, weaning weight was included as a co-variate. For BW at slaughter and cold carcass weight, the number of days from weaning to slaughter was included as a co-variate. Day was included in the above models as a repeated variable when relevant. Pre-weaning block was included as a random effect for the analysis of all pre-weaning parameters. Post-weaning block was included as a random effect for the analysis of all post-weaning growth parameters. The litter/sow was the experimental unit for the analysis of all pre-weaning parameters, except piglet weight and growth, where pig nested within sow/litter was the experimental unit. The experimental unit post-weaning was the pen group.

For analysis of intestinal morphology and blood parameters, treatment was included in the model as a fixed effect. Sow was included as a random effect and the pig was the experimental unit.

In all cases, differences in least square means were investigated using the t test after Tukey adjustment for multiple comparisons. Results are presented in the text and tables as the least square means together with their pooled standard error. Differences between treatments were considered significant when *P* ≤ 0.05, whereas 0.05 < *P* ≤ 0.10 was considered as a tendency.

## RESULTS

### Sow Body Weight and Back Fat Thickness

The effect of treatment on sow BW and BF depth from farrowing to service (~4 d after weaning) is presented in Table S3. There was no effect of treatment (*P* > 0.05) on any parameter of interest at any time point.

### Mortality and Removals

The effect of treatment on sow litter size at day 8 and weaning and the number of piglet deaths per litter from day 8 to weaning is presented in Table S4. There was no effect of treatment on any parameter of interest (*P* > 0.05).

### Pre-Weaning Pig Growth Performance

The effect of treatment on piglet creep feed DMd, pig weight and growth during the suckling period is presented in Table 2. There was no treatment effect on pre-weaning DMd of creep feed per pig, net energy intake per pig per day, lysine intake per pig per day, L-glutamine intake per pig per day or on litter weight at any time point from day 8 to weaning at day 28 (*P* > 0.05). There was no effect of treatment on mean piglet BW at day 8, 14 and overall (*P* > 0.05) during lactation. L-glutamine supplementation tended to decrease mean piglet BW at day 21 (*P* = 0.09) and day 28 (*P* = 0.08). There was no effect of treatment on ADG from day 8 to 14, day 21 to 27 and overall during lactation (*P* > 0.05). Piglets supplemented with L-glutamine had a lower ADG from day 14 to 21 than control piglets and piglets supplemented with enzymes (*P* < 0.01).

**Table 2.** Effect of dietary treatment on creep feed dry matter disappearance, litter weight, piglet weight and growth during the suckling period

Treatment	Control	Glutamine	Enzymes	SEM	*P*-value
Number of litters	21	20	19		
**Litter weight, kg**					
Day 8	42.0	42.3	40.7	1.46	0.42
Day 14	64.0	64.3	62.9	1.49	0.55
Day 21	90.8	88.4	89.1	1.89	0.52
Day 27	114.1	109.5	111.7	2.54	0.35
Overall	77.7	76.1	76.1	1.38	0.22
**Mean piglet BW, kg** ^1^					
Day 8	3.10	3.09	3.12	0.060	0.88
Day 14	4.73	4.72	4.76	0.060	0.88
Day 21	6.70^A^	6.57^B^	6.69^A^	0.060	0.09
Day 27	8.41^A^	8.28^B^	8.41^A^	0.060	0.08
Overall				0.055	0.32
**ADG, g/day** ^2^					
Day 8 to 14	260	259	260	5.8	0.96
Day 14 to 21	280^a^	261^b^	277^a^	6.5	<0.01
Day 21 to 27	283	280	288	7.2	0.58
Overall	274	267	275	5.7	0.13
**Dry matter disappearance**					
Pre-weaning creep feed, g/pig	202	224	197	25.9	0.77
Net energy intake from day 8 to 27, MJ/pig/day	0.13	0.14	0.13	0.017	0.78
Lysine intake from day 8 to 27, g/pig/day	0.18	0.19	0.17	0.023	0.78
L-glutamine intake from day 8 to 27, g/pig/day	0.11	0.12	0.11	0.014	0.78

^1^BW, body weight.

^2^ADG, average daily gain.

Values within a row that do not share a common superscript are significantly different (*P* ≤ 0.05).

^A, B^Values within a row that do not share a common superscript tended to differ (0·05 < *P* ≤ 0·10).

Control, liquid starter diet provided as creep feed from day 8 of age to weaning; Glutamine, control supplemented with 10 g of L-glutamine per kg of starter diet; Enzymes, control supplemented with a cocktail of enzymes (lipase, protease and α-amylase).

### Live Observation of Feeder-Directed Activity Behavior Per Litter

The proportion of piglets having two or more feeder-directed activities on each day of live observation and overall within a litter is presented in Table S5. There was no effect of treatment on the percentage of eaters at day 13, 16 and 22. Overall, piglets supplemented with enzymes tended to have a higher percentage of eaters than control piglets and piglets supplemented with L-glutamine (*P* = 0.07). The proportion of piglets having one or more feeder-directed activities on each day of live observation and overall within a litter is presented in Table S6. At day 16 of lactation and overall, piglets supplemented with enzymes tended to have a higher percentage of eaters than control piglets and piglets supplemented with L-glutamine (*P* = 0.09 and *P* = 0.06, respectively).

### Pre-Weaning Diarrhea Scores, Clinical Cases of Disease and Antibiotic and Anti-Inflammatory Treatment

The effect of treatment on diarrhea prevalence from day 8 of age to weaning is presented in Table 3. L-glutamine supplementation tended to increase diarrhea prevalence in piglets (*P* = 0.09). The effect of treatment on the total number of clinical cases of disease per litter, the total number of injections per litter, and the average amount of medication (antibiotic and anti-inflammatory) administered per pig on a litter basis, and per sow, during the pre-weaning period is presented in Table 3. There was no effect of treatment on any of these parameters (*P* > 0.05).

**Table 3.** Effect of dietary treatment on health parameters and medicinal treatment of sows and piglets during lactation

Treatment	Control	Glutamine	Enzymes	SEM	*P*-value
**Number of sows**	21	20	19		
Diarrhea prevalence, % (day 8 to 28)^1^	11^B^	23^A^	12^B^	0.4	0.09
No. of clinical cases of disease per litter^2^	1.3	1.1	0.8	0.42	0.76
No. of injections per litter	3.8	3.2	2.4	1.28	0.76
Antibiotic usage per sow, mL^3^	0	7.2	2.5	2.66	0.16
Antibiotic usage per pig, mL^4^	0.13	0.12	0.09	0.045	0.82
Anti-inflammatory usage per sow, mL^5^	0	1.8	0.6	0.66	0.16
Anti-inflammatory usage per pig, mL^6^	0.03	0.02	0.02	0.009	0.79

^1^A fecal score of 2 or greater for a litter was considered indicative of diarrhea at each time point between day 8 and 28. The overall prevalence was reported for the pre-weaning period.

^2^Number of piglets per litter treated one or more times.

^3^Amount of antibiotic administered per sow.

^4^Amount of antibiotic administered per piglet.

^5^Amount of anti-inflammatory administered per sow.

^6^Amount of anti-inflammatory administered per piglet.

^A, B ^Values within a row that do not share a common superscript tended to differ (0·05 < *P* ≤ 0·10). Control, liquid starter diet provided as creep feed from day 8 of age to weaning; Glutamine, control supplemented with 10 g of L-glutamine per kg of starter diet; Enzymes, control supplemented with a cocktail of enzymes (lipase, protease and α-amylase).

### Post-Weaning Pig Growth and Carcass Quality

The effect of treatment on pig growth, feed intake and feed efficiency from weaning to slaughter is presented in Table 4. There was no effect of treatment on pig ADFI or BW at any time point post-weaning (*P* > 0.05). Pigs supplemented with L-glutamine pre-weaning tended to have better ADG from day 21 to 28 post-weaning than pigs supplemented with enzymes pre-weaning (*P* = 0.07). There was no effect of treatment on ADG at any other time point (*P* > 0.05). Pigs supplemented with L-glutamine pre-weaning tended to have higher G:F from day 21 to 28 post-weaning than pigs supplemented with enzymes pre-weaning (*P* = 0.07). There was no effect of treatment on G:F at any other time point (*P* > 0.05).

**Table 4.** Effect of dietary treatment on feed intake and growth of pigs from weaning to slaughter at ~120 kg

Treatment	Control	Glutamine	Enzymes	SEM	*P*-value
Days from weaning to slaughter	132	132	132		
**BW, kg** ^1^					
Day 0 (weaning)	8.5	8.3	8.0	1.54	0.98
Day 6 post-weaning	9.9	9.7	9.6	1.54	0.99
Day 14 post-weaning	12.9	12.6	12.6	1.54	0.99
Day 21 post-weaning	16.5	16.2	16.2	1.54	0.99
Day 28 post-weaning	20.9	21.0	20.5	1.54	0.97
Day 43 post-weaning	33.3	33.2	32.3	0.52	0.35
Day of slaughter (~158 d of age)	134.0	136.5	132.6	2.08	0.42
Overall				0.54	0.73
**ADFI, g/day** ^2^					
Day 0 to 6	240	244	252	11.2	0.60
Day 6 to 14	501	491	456	22.5	0.33
Day 14 to 21	638	644	630	34.8	0.96
Day 21 to 28	906	892	896	37.4	0.96
Day 28 to 47	1258	1284	1262	60.4	0.94
Day 43 to slaughter	2783	2738	2817	36.8	0.33
Overall				25.5	0.94
**ADG, g/day** ^3^					
Day 0 to 6	253	227	250	23.1	0.59
Day 6 to 14	390	371	371	25.0	0.80
Day 14 to 21	529	522	498	22.6	0.54
Day 21 to 28	643^A,B^	689^A^	598^B^	28.2	0.07
Day 28 to 43	829	815	800	24.3	0.75
Day 43 to slaughter	1139	1160	1123	27.0	0.61
Overall				17.8	0.44
**G:F** ^4^ **, g/g**					
Day 0 to 6	1.04	0.94	1.01	0.033	0.11
Day 6 to 14	0.77	0.75	0.83	0.033	0.27
Day 14 to 21	0.82	0.81	0.80	0.033	0.92
Day 21 to 28	0.70^A,B^	0.77^A^	0.67^B^	0.033	0.07
Day 28 to 43	0.65	0.64	0.64	0.033	0.99
Day 43 to slaughter	0.40	0.42	0.40	0.033	0.88
Overall				0.729	0.94

^1^BW, body weight.

^2^ADFI, average daily feed intake.

^3^ADG, average daily gain.

^4^G:F, gain to feed ratio.

^A, B^ Values within a row that do not share a common superscript tended to differ (0·05 < *P* ≤ 0.10). Only 6 pen replicates by treatment could be used to analyze the growth performance data up to day 43 post-weaning. Data from day 43 post-weaning to slaughter day were analyzed using 12 pen replicates by treatment.

Control, liquid starter diet provided as creep feed from day 8 of age to weaning; Glutamine, control supplemented with 10 g of L-glutamine per kg of starter diet; Enzymes, control supplemented with a cocktail of enzymes (lipase, protease and α-amylase).

The effect of treatment on carcass parameters is presented in Table S7. There was no effect of treatment on any parameter of interest (*P* > 0.05).

### Post-Weaning Diarrhea Scores, Antibiotic and Anti-Inflammatory Treatment

The effect of treatment on diarrhea prevalence from weaning to day 28 post-weaning is presented in Table S8. There was no effect of treatment on diarrhea prevalence between weaning and day 28 post-weaning (*P* > 0.05).

The effect of treatment on the average amount of medication (antibiotic and anti-inflammatory) administered per pig on a pen basis and the number of clinical cases of disease per pen post-weaning is presented in Table S8. There was no effect of treatment on any of these parameters during the weaner or finisher periods.

### Haematology

The effect of treatment on haematological parameters of pigs at day 5 post-weaning is presented in Table 5. L-glutamine supplementation pre-weaning increased the red blood cell count (*P* = 0.03) and hemoglobin concentration (*P* = 0.04) in the plasma of pigs at day 5 post-weaning compared to control and enzymes. There was no effect of treatment on any other parameter of interest (*P* > 0.05).

**Table 5.** Effect of dietary treatment on haematological parameters of weaned pigs at day 5 post-weaning.

Treatment	Control	Glutamine	Enzymes	SEM	P-value	Reference ranges^1^
Number of pigs	10	10	10			
White blood cells (× 10^3^ cells/μl)	8.53	7.23	7.49	0.94	0.60	9.62 to 25.2
Lymphocytes (× 10^3^ cells/µL)	2.99	2.89	2.47	0.459	0.70	4.02 to 12.5
Monocytes (× 10^3^ cells/µL)	0.61	0.56	0.53	0.119	0.88	0.05 to 2.3
Neutrophils (× 10^3^ cells/µL)	4.72	3.62	4.36	0.639	0.47	2.35 to 11.9
Eosinophils (× 10^3^ cells/µL)	0.14	0.12	0.08	0.025	0.17	0 to 0.05
Basophils (× 10^3^ cells/µL)	0.05	0.06	0.06	0.015	0.80	−
Red blood cells (× 10^6^ cells/µL)	4.98^b^	5.40^a^	4.56^b^	0.212	0.03	4.87 to 7.88
Haemoglobin (g/dL)	8.90^b^	9.72^a^	8.22^b^	0.402	0.04	8.08 to 11.9
Mean corpuscular F (fL)	61.1	60.6	60.7	1.03	0.94	43.3 to 64.5
Mean corpuscular hemoglobin (pg/cell)	17.9	18.0	18.0	0.29	0.95	12.4 to 19.3
Mean corpuscular hemoglobin concentration (g/dL)	29.3	29.7	29.7	0.32	0.58	27.3 to 31.4
Platelets (× 10^3^ cells/μL)	249	317	216	46.9	0.33	374.3 to 1080.8

^1^Normal reference ranges for pigs 0 to 6 wk old (Iowa State University, 2011).

^a,b^ Values within a row that do not share a common superscript differ significantly at P < 0.05.

^A,B^ Values within a row that do not share a common superscript tended to differ at 0.05 < P ≤ 0.10.

Control, liquid starter diet provided as creep feed from day 8 of age to weaning; Glutamine, control supplemented with 10 g of L-glutamine per kg of starter diet; Enzymes, control supplemented with a cocktail of enzymes (lipase, protease and α-amylase).

### Small Intestinal Histology

The effect of treatment on small intestinal morphology of pigs at day 5 post-weaning is presented in Table S9. There was no effect of treatment on VH, CD, VH to crypt depth ratio (VH:CD) or villus width in the duodenum, jejunum and ileum of pigs (*P* > 0.05).

## DISCUSSION

To our knowledge, this study is the first to determine the effect of creep feeding a liquid starter diet with or without the inclusion of L-glutamine or a cocktail of enzymes to suckling pigs on pre- and post-weaning growth to target slaughter weight (~120 kg), health and intestinal structure.

### L-glutamine

In the current study, the decision to use L-glutamine in the starter diet at an inclusion rate of 1% was based on a study conducted by Cabrera et al. (2013) with healthy pigs. Cabrera et al. (2013) found an improvement in feed efficiency (early post-weaning), intestinal epithelial cell proliferation and villus height at 7 d post-weaning when 1% L-glutamine was included in the creep feed and in the diet fed for 6 wk post-weaning. They observed a creep feed intake of ~46 g/pig when supplemented 1 wk prior to weaning. Therefore, piglets ingested 0.06 g of L-glutamine/day before weaning. Based on this, we would have expected a 1% L-glutamine inclusion rate in the creep feed of the current study to give a similar response. However, supplementing pigs with L-glutamine in liquid creep feed tended to reduce pig weight at weaning and to increase pre-weaning diarrhea prevalence. There are no previous reports linking glutamine supplementation of pig diets with increased diarrhea prevalence. Glutamine was previously shown to positively influence pig BW when supplemented orally to lipopolysaccharide-challenged piglets pre-weaning at 1 g/kg of pig BW per day but to be detrimental for piglet BW when supplemented at 2 g/kg of pig BW per day (Haynes et al., 2009). In the current study DMd was 224 g/pig during the period of supplementation and L-glutamine was included in the diet at 10 g/kg fresh weight (i.e., 1%). Therefore, piglets theoretically ingested ~ 2.42 g of L-glutamine in total between day 8 and weaning, which corresponds to ~0.12 g of L-glutamine/pig/day or ~0.02 g/kg of pig mean BW during supplementation. This is well below the dose considered efficacious by Haynes et al. (2009). Creep feed DMd was lower in the current study than that reported in other studies when liquid feed was supplemented to suckling piglets (Kobek-Kjeldager et al., 2021; Vasa et al., 2023). We would have expected the total creep feed DMd to be closer to 500 g/pig as in our previous study (Vasa et al., 2023), which would have resulted in an L-glutamine intake of ~0.27 g/pig/day (~0.05 g/kg of mean pig BW during supplementation). This is still lower by a factor of 20 compared to the 1g/kg of pig BW previously shown to be effective by Haynes et al. (2009).

The level of free glutamine in sows’ milk increases during the lactation period from ~0.18 g/L at day 8 to ~0.50 g/L at day 29 (Wu et al., 2011). Between 3 and 4 wk of age, a pig with a weaning weight of 8 kg ingests up to 2 L of sow’s milk per day (Wu et al., 2011). Therefore, a pig at this time has a glutamine intake of 1 g/day from sow’s milk or ~0.13 g/kg of pig BW (Wu et al., 2011). Hence, based on this data, the L-glutamine level provided to suckling piglets in the current study, even with that supplied from sow’s milk, was well below the optimum suggested in the study by Haynes et al. (2009).

Furthermore, although free glutamine was found to be present in the dry feed at the 1% inclusion level, free glutamine was only found at <0.2 g/kg DM in the liquid starter diet (after oven drying). This was unexpected since the liquid diets were prepared freshly every 6 hours and L-glutamine had previously been shown to remain stable in water for 24 hours (Molfino et al., 2009). Likewise, oven drying the liquid creep diet at 55 °C, prior to analysis, was not expected to affect its stability, since previous work demonstrated that it remained stable after feed pelleting at 70 to 88 °C (Bampidis et al., 2020). However, in liquid feed, nutrients and energy can be used during microbial fermentation, leading to significant amino acid and gross energy losses from the feed (Cullen et al., 2021; O’Meara et al., 2021). Decarboxylase enzymes can be produced by various microbes, including lactic acid bacteria and microbial decarboxylation of amino acids in particular can occur under acidic conditions (Barbieri et al., 2019; Yazgan et al., 2021). In a previous study, we demonstrated that lactic acid bacteria grew by ~2 log_10_ colony forming units per mL in liquid feed during the 6-h period between preparation and the last feed of the day (Arnaud et al., 2024). This likely explains the reduced level of free glutamine observed in the liquid creep feed. Another issue is that microbial decarboxylation of amino acids can result in the production of undesirable metabolites, such as biogenic amines (Barbieri et al., 2019). Microbial decarboxylation of free glutamine can result in the production of ornithine, and two biogenic amines, cadaverine and putrescine, are subsequently formed from the decarboxylation of ornithine (Özogul and Özogul, 2019).These biogenic amines are known to reduce feed palatability and can reduce feed intake in pigs. They are also cytotoxic at high doses (Del Rio et al., 2019). This might explain the tendency for L-glutamine to reduce piglet growth and increase diarrhea prevalence pre-weaning in the current study. Therefore, it is likely that liquid creep feed is not a good vehicle for L-glutamine.

Weaning is often associated with reduced feed intake in pigs (Lawlor et al., 2020). The lack of nutrient supply at this time can negatively affect integrity of the intestinal tract, and therefore reduce nutrient absorption and feed efficiency (Tang et al., 2022). There was no improvement in small intestinal morphology at day 5 post-weaning in response to pre-weaning L-glutamine supplementation. Pre-weaning L-glutamine supplementation increased the red blood cell count and hemoglobin concentration in plasma of pigs on day 5 post-weaning. This agrees with the literature, as Dumaswala et al. (1994) demonstrated that glutamine is important for the preservation of red blood cells. It plays an important role in the regulation of oxidative stress which makes its use of interest in the case of red blood cell disorders (e.g., sickle cell anemia in humans) (Elenga et al., 2022). In addition, Bhattarai and Nielsen (2015) found a positive association between both hemoglobin concentration and red blood cell count and ADG in pigs post-weaning. However, it is important to note that in the current study, red blood cell counts and hemoglobin concentrations in the plasma of both unsupplemented and supplemented pigs were within the normal ranges for weaned pigs and no improvement in ADG was observed in response to L-Glutamine supplementation. Most haematological parameters were within normal reference ranges, with the exception of white blood cell, lymphocyte and platelet counts which were lower and eosinophil count which was higher than the normal ranges for weaned pigs (Iowa State University, 2011). The experimental pig facility, where the current study was performed was of high health status and observed strict internal and external biosecurity. Consequently, it would be expected to have very low levels of disease pressure compared with commercial farms. This likely explains the lower than normal counts of white blood cells and lymphocytes observed, as these immune cells are solicited and activated during the establishment of an immune response.

### Enzymes

Supplementing suckling pigs with the cocktail of enzymes in liquid creep feed did not affect pig weaning weight. This is in agreement with the findings of Prykhodko et al. (2016) who gavage-fed suckling pigs a mixture of amylase, protease and lipase. However, it was surprising that, post-weaning growth and feed efficiency were also not affected by the pre-weaning treatment in the current study, as Prykhodko et al. (2016) did find increased growth during the grow-finisher period. Słupecka et al. (2012) previously showed that adequate supplementation of pancreatic enzymes to pigs during the postnatal period enhanced digestive tract development in piglets. This would be expected to improve post-weaning growth performance as demonstrated by Prykhodko et al. (2016). Although not measured, it would seem unlikely that the development of inherent enzyme production capacity in pigs at weaning was increased in the current study in response to pre-weaning supplementation with the extraneous enzyme cocktail.

In addition, enzyme supplementation did not affect intestinal morphology at day 5 post-weaning. Contrary to this, Słupecka et al. (2012) observed that supplementing suckling piglets with porcine pancreatic enzymes twice a day from day 8 to 15 of age by oral gavage increased villus height and reduced crypt depth in the jejunum. It could be that piglets in the present study did not ingest sufficient amounts of the enzyme cocktail. However, feed analyses found that both the protease and the α-amylase were recovered at expected concentrations from the dry and dried liquid starter diet. It was not possible to determine the recovery of lipase from the diets, as the methodology has yet to be developed by the enzyme supplier. Total creep feed DMd (197 g of DM/pig of creep feed) as well as the overall percentage of piglets considered as eaters (20%) were both very low in the current study. Based on our previous work with liquid creep (Vasa et al., 2023), DMd was expected to be twice that recorded here. The lower DMd observed in the current study is most likely because liquid feed was prepared with water as opposed to liquid milk replacer in the case of Vasa et al. (2023). It could be speculated that the low DMd found in the present study, meant that piglets did not ingest a sufficient quantity of each enzyme to positively influence intestinal structure and function and post-weaning growth. If insufficient creep feed intake and hence, insufficient enzyme intake is the reason for the lack of response to enzyme supplementation in the current study, then creep feed DMd and consequently enzyme intake could certainly be increased in future studies by preparing the liquid feed with a liquid milk replacer, as in our previous studies (Vasa et al., 2023; Arnaud et al., 2024). However, the lack of response to enzyme supplementation in the current study could also be due to the choice of enzymes or their route of administration. In the present study, a lipase, an α-amylase and a protease of microbial origin were selected, as supplementation of these microbially-derived enzymes pre-weaning had previously been shown to increase feed efficiency and growth in pigs post-weaning (Prykhodko et al., 2016). In the current study, the lipase was of fungal origin (*Rhizopus*) and the α-amylase and proteases were of bacterial origin (*Bacillus*). In the study conducted by Prykhodko et al. (2016), the lipase and protease were of fungal origin (*Aspergillus*) and the amylase of bacterial origin (*Burkholderia*). These differences could explain the lack of response to enzymes in the current study. In addition, to the author’s knowledge, this study was the first where a cocktail of enzymes was administered to the piglets via creep feed and not by gavage. Supplementing pigs by gavage ensures that all of the pigs receive the intended amount of the enzyme cocktail in contrast to the creep feed supplementation employed in the current study. This could therefore, also explain the lack of response to enzymes in the current study.

## CONCLUSIONS

Suckling piglets offered liquid creep feed supplemented with L-glutamine tended to have an increased prevalence of pre-weaning diarrhea and lighter weaning weight. Supplementing liquid creep feed with an enzyme cocktail had no effect on growth, health or medication usage in pigs. The intestinal morphology of pigs at day 5 post-weaning was not affected by pre-weaning supplementation with L-glutamine or the enzyme cocktail. It may be that an insufficient level of supplementation with L-glutamine combined with lower than expected creep feed intake and likely degradation of L-glutamine in the liquid creep feed are responsible for the absence of a response to L-glutamine in this study. The lack of response to the enzyme cocktail could be due to the low creep feed intake, the fact that the enzymes originated from different microbial strains than enzymes previously shown to be effective or it could be due to their route of administration (creep feed vs. gavage). Consideration should be given to feeding liquid milk replacer or a mixture of milk replacer and starter diet in future studies to ensure high liquid creep feed intakes and as a consequence increased feed additive consumption. A combination of liquid and dry creep feed should also be considered in future studies.

## Supplementary Data

Supplementary data are available at *Translational Animal Science* online.

txaf066_suppl_Supplementary_Tables_S1-S9
