# Supplementary material for: L-glutamine- and enzyme-supplementation via liquid feed to suckling piglets does not impact growth, health or intestinal structure
Source: Transl Anim Sci. 2025 May 16;9:txaf066. doi: 10.1093/tas/txaf066 (PMC12125623; doi:10.1093/tas/txaf066)
Supplement: txaf066_suppl_Supplementary_Tables_S1-S9 [file txaf066_suppl_supplementary_tables_s1-s9.docx]

**Supplementary Tables**

**Table S1. L-glutamine, glutamic acid and enzyme recovery analyses on the dry pelleted starter diet and on oven dried liquid starter diet.**

| **Additives** | **Dry starter control** | **Dry starter glutamine** | **Dry starter**  **enzymes** | **Dried liquid**  **starter control^1^** | **Dried liquid**  **starter glutamine^1^** | **Dried liquid**  **starter enzymes^1^** |
| --- | --- | --- | --- | --- | --- | --- |
| **Amino acids** |  |  |  |  |  |  |
| Glutamine, % (free) | <0.02 | 0.858 | <0.02 | <0.02 | <0.02 | <0.02 |
| Glutamic acid, % (free) | 0.04 | 0.05 | 0.04 | 0.04 | 0.24 | 0.10 |
| Total glutamic acid, % | 3.21 | 4.09 | 3.22 | 3.28 | 3.79 | 3.39 |
| **Enzymes** |  |  |  |  |  |  |
| Protease, NFP/kg^2^ | <LOD | <LOD | 40650 | <LOD | <LOD | 12860 |
| α-amylase, KNU/kg^3^ | <LOD | <LOD | 67 | <LOD | <LOD | 52 |

**^1^**The liquid starter diet samples prepared by mixing dry pelleted starter diet with warm water (55 ˚C) at a ratio of 1:5 and incubated for 5 hours at 30°C. Before conducting these analyses, the liquid starter diet was oven dried at 55 ˚C for 3 consecutive days.

^2^ProAct 360 (DSM-Firmenich, Heerlen, The Netherlands); NFP, New Feed Protease units

^3^RONOZYME HiStarch (DSM-Firmenich); KNU, Kilo Novozymes units

LOD, Limit of detection

| **Response variable** | **Covariate** | **Co-variance structure** |
| --- | --- | --- |
| Sow body weight | Sow body weight at day 108 of gestation | AR(1) |
| Sow back fat | Sow back fat at day 108 of gestation | AR(1) |
| Piglet body weight pre-weaning | Litter size at day 8 pre-weaning  Body weight at day 8 pre-weaning | ARH(1) |
| Piglet average daily gain pre-weaning | Litter size at day 8 pre-weaning  Body weight at day 8 pre-weaning | AR(1) |
| Pig body weight post-weaning | Weaning body weight | AR(1) |
| Pig body weight at slaughter | Weaning body weight | AR(1) |
| Pig carcass weight | Weaning body weight | CS |

**Table S2. Covariance structure chosen using the lowest Akaike Information Criteria.**

**Table S3. Effect of treatment on sow body weight and back-fat depth.**

| **Treatment^1^** | **Control** | **Glutamine** | **Enzymes** | **SEM** | **P-value** |
| --- | --- | --- | --- | --- | --- |
| Number of sows | 21 | 20 | 19 |  |  |
| Lactation length, days | 27.6 | 27.7 | 27.8 | 0.79 |  |
| **BW, kg^1^** |  |  |  |  |  |
| Day 108 of gestation | 284 | 284 | 284 | 2.4 | 0.99 |
| Farrowing^2^ | 248 | 247 | 246 | 2.4 | 0.88 |
| Weaning^3^ | 240 | 241 | 242 | 2.4 | 0.76 |
| Service^3^ | 232 | 231 | 234 | 3.0 | 0.79 |
| Overall |  |  |  | 1.8 | 0.92 |
| **BF, mm^4^** |  |  |  |  |  |
| Day 108 of gestation | 18.4 | 18.2 | 18.5 | 0.29 | 0.86 |
| Weaning | 14.0 | 14.3 | 13.7 | 0.29 | 0.36 |
| Service | 14.3 | 14.6 | 13.9 | 0.36 | 0.29 |
| Overall |  |  |  | 0.22 | 0.48 |
| **Sow BW change, kg** |  |  |  |  |  |
| Day 108 to weaning^5^ | -43 | -42 | -41 | 3.0 | 0.81 |
| Farrowing to weaning^6^ | -7 | -6 | -1 | 3.0 | 0.25 |
| Weaning to service^7^ | -13 | -8 | -4 | 4.0 | 0.20 |
| Overall |  |  |  | 2.3 | 0.06 |
| **Sow BF change, mm** |  |  |  |  |  |
| Day 108 to weaning^8^ | -4.4 | -3.9 | -4.8 | 0.34 | 0.21 |
| Weaning to service^9^ | 0.2 | 0.6 | 0.3 | 0.45 | 0.83 |
| Overall |  |  |  | 0.29 | 0.35 |

^1^BW, body weight.

^2^Estimated empty farrowing weight = (sow weight at day 108 – (total born × 2.25)). The value of 2.25 kg is an estimate of the increased weight in the gravid uterus and in mammary tissue attributed to each pig in a litter (NRC, 1998).

^3^Weaning = day 28 ± 1 of lactation; service = day 4 ± 1 post-weaning.

^4^BF, back fat.

^5^Sow BW change = (sow BW at weaning – sow BW at day 108 of gestation).

^6^Sow BW change = (sow BW at weaning – sow BW at farrowing).

^7^Sow BW change = (sow BW at service – sow BW at weaning).

^8^Sow BF change = (sow BF at weaning – sow BF at day 108 of gestation).

^9^Sow BF change = (sow BF at service – sow BF at weaning).

Control, liquid starter diet provided as creep feed from day 8 of age to weaning; Glutamine, control supplemented with 10 kg of L-glutamine per tonne of starter diet; Enzymes, control supplemented with a cocktail of enzymes (lipase, protease and α-amylase)

**Table S4. Effect of treatment on sow litter size and the number of piglets fostered and died per litter during lactation.**

| **Treatment** | **Control** | **Glutamine** | **Enzymes** | **SEM** | **P-value** |
| --- | --- | --- | --- | --- | --- |
| Number of sows | 21 | 20 | 19 |  |  |
| **Litter** |  |  |  |  |  |
| Litter size at day 8 | 14.0 | 13.6 | 13.8 | 0.40 | 0.72 |
| Litter size weaning | 13.9 | 13.2 | 13.6 | 0.41 | 0.46 |
| **Deaths and removals per sow** |  |  |  |  |  |
| Deaths after day 8 | 0.1 | 0.4 | 0.3 | 0.13 | 0.37 |

Control, liquid starter diet provided as creep feed from day 8 of age to weaning; Glutamine, control supplemented with 10 kg of L-glutamine per tonne of starter diet; Enzymes, control supplemented with a cocktail of enzymes (lipase, protease and α-amylase)

**Table S5.** **Effect of treatment on percentage of “eaters” within a litter where two or more feeder-directed activities per pig is considered to indicate an “eater”.**

| **Treatment** | **Control** | **Glutamine** | **Enzymes** | **SEM** | **P-value** |
| --- | --- | --- | --- | --- | --- |
| **Eaters, %^1^** |  |  |  |  |  |
| Day 13 of lactation | 10 | 15 | 19 | 5.8 | 0.47 |
| Day 16 of lactation | 11 | 7 | 21 | 5.8 | 0.12 |
| Day 22 of lactation | 12 | 10 | 18 | 5.8 | 0.50 |
| Overall | 11^B^ | 11^B^ | 20^A^ | 4.0 | 0.07 |

^1^An eater was defined as having two or more feeder trough-directed-activity observations on each day. The percentage of piglet eaters per pen was calculated on a pen basis for each observation day and for all observation days combined. This was carried out by expressing the number of piglets considered as eaters in a pen as a percentage of the total number of piglets present in the pen.

^A, B^ Values within a row that do not share a common superscript tended to differ (0.05<*P*≤0.10).

Control, liquid starter diet provided as creep feed from day 8 of age to weaning; Glutamine, control supplemented with 10 kg of L-glutamine per tonne of starter diet; Enzymes, control supplemented with a cocktail of enzymes (lipase, protease and α-amylase)

**Table S6.** **Effect of treatment on percentage of “eaters” within a litter where one or more feeder-directed activities per pig is considered to indicate an “eater”.**

| **Treatment** | **Control** | **Glutamine** | **Enzymes** | **SEM** | **P-value** |
| --- | --- | --- | --- | --- | --- |
| **Eaters, %^1^** |  |  |  |  |  |
| Day 13 of lactation | 31 | 36 | 49 | 8.5 | 0.30 |
| Day 16 of lactation | 26^B^ | 26^B^ | 49^A^ | 8.5 | 0.09 |
| Day 22 of lactation | 40 | 32 | 41 | 8.5 | 0.70 |
| Overall | 32^B^ | 31^B^ | 46^A^ | 5.3 | 0.06 |

^1^An eater was defined as having one or more feeder trough-directed-activity observations on each day. The percentage of piglet eaters per pen was calculated on a pen basis for each observation day and for all observation days combined. This was carried out by expressing the number of piglets considered as eaters in a pen as a percentage of the total number of piglets present in the pen.

^A, B^ Values within a row that do not share a common superscript tended to differ (0.05<*P*≤0.10).

Control, liquid starter diet provided as creep feed from day 8 of age to weaning; Glutamine, control supplemented with 10 kg of L-glutamine per tonne of starter diet; Enzymes, control supplemented with a cocktail of enzymes (lipase, protease and α-amylase)

**Table S7. Effect of dietary treatment on pig carcass parameters at slaughter**

| **Treatment** | **Control** | **Glutamine** | **Enzymes** | **SEM** | ***P*-value** |
| --- | --- | --- | --- | --- | --- |
| Number of pens | 12 | 12 | 12 |  |  |
| Cold carcass weight, kg | 102.8 | 104.2 | 102.6 | 1.09 | 0.50 |
| Fat depth, mm | 14.7 | 14.7 | 14.9 | 0.35 | 0.90 |
| Muscle depth, mm | 53.3 | 52.3 | 54.0 | 0.74 | 0.30 |
| Lean meat, % | 57.6 | 57.5 | 57.5 | 0.27 | 0.96 |
| Kill out, % | 76.8 | 76.3 | 77.7 | 0.89 | 0.57 |
| Carcass ADG, g/d^1,2^ | 741 | 751 | 747 | 10.4 | 0.80 |
| Carcass G:F, g/g^3,4^ | 0.33 | 0.34 | 0.33 | 0.004 | 0.13 |
| Lean ADG, g/d^5^ | 373 | 377 | 376 | 4.95 | 0.83 |

^1^ADG, average daily gain.

^2^Carcass ADG (from weaning to slaughter) = [(carcass weight in kg –weaning weight in kg × 0.55) × 1,000]/number of days from weaning to slaughter (Lawlor and Lynch, 2005).

^3^G:F,gain to feed ratio.

^4^Carcass G:F (from weaning to slaughter) was calculated as follows: carcass G:F = carcass ADG (g)/ daily feed intake (g).

^5^Lean ADG (from birth to slaughter) = (carcass weight × carcass lean meat percentage × 10)/number of days to slaughter (Lawlor and Lynch, 2005)

Control, liquid starter diet provided as creep feed from day 8 of age to weaning; Glutamine, control supplemented with 10 g of L-glutamine per kg of starter diet; Enzymes, control supplemented with a cocktail of enzymes (lipase, protease and α-amylase)

**Table S8. Effect of dietary treatment on post-weaning diarrhoea prevalence and medicinal treatment of pigs**

| **Treatment** | **Control** | **Glutamine** | **enzymes** | **SEM** | ***P*-value** |
| --- | --- | --- | --- | --- | --- |
| Number of pens | 12 | 12 | 12 |  |  |
| Diarrhoea prevalence, % (weaning to 28 days post-weaning)^1^ | 23 | 22 | 23 | 0.1 | 0.97 |
| **Weaner period (weaning to day 43 post-weaning)** |  |  |  |  |  |
| No. of clinical cases of disease per pen^2^ | 1.1 | 0.3 | 0.4 | 0.37 | 0.24 |
| Antibiotic usage per pig, mL^3^ | 0.33 | 0.08 | 0.13 | 0.112 | 0.24 |
| Anti-inflammatory usage per pig, mL^4^ | 0.18 | 0.05 | 0.08 | 0.059 | 0.25 |
| **Finisher period (day 43 to day 158 post-weaning)** |  |  |  |  |  |
| No. of clinical cases of disease per pen^2^ | 0.2 | 0.3 | 0.2 | 0.15 | 0.91 |
| Antibiotic usage per pig, mL^3^ | 0.25 | 0.45 | 0.27 | 0.241 | 0.81 |
| Anti-inflammatory usage per pig, mL^4^ | 0.05 | 0.08 | 0.06 | 0.043 | 0.91 |

^1^A faecal score of 2 or greater was considered indicative of diarrhoea at each time-point (day 1, 5, 14, 21 and 28 post-weaning) between weaning and day 28 post-weaning. The overall prevalence was reported for the post-weaning period.

^2^Number of pigs per pen treated one or more times.

^3^ Amount of antibiotic administered to each pig on a pen basis.

^4^ Amount of anti-inflammatory administered to each pig on a pen basis.

Control, liquid starter diet provided as creep feed from day 8 of age to weaning; Glutamine, control supplemented with 10 kg of L-glutamine per tonne of starter diet; Enzymes, control supplemented with a cocktail of enzymes (lipase, protease and α-amylase)

**Table S9. Effect of dietary treatment on small intestinal morphology of pigs at day 5 post-weaning**

| **Treatment** | **Control** | **Glutamine** | **Enzymes** | **SEM** | ***P*-value** |
| --- | --- | --- | --- | --- | --- |
| Number of pigs |  |  |  |  |  |
| **Duodenum** |  |  |  |  |  |
| Villus height (μm) | 357 | 314 | 331 | 24.5 | 0.46 |
| Crypt depth (μm) | 169 | 184 | 200 | 12.4 | 0.23 |
| VH:CD^1^ ratio (μm/μm) | 2.54 | 2.14 | 2.09 | 0.188 | 0.19 |
| Villus width (μm) | 150 | 134 | 143 | 5.8 | 0.18 |
| **Jejunum** |  |  |  |  |  |
| Villus height (μm) | 269 | 242 | 300 | 18.6 | 0.11 |
| Crypt depth (μm) | 166 | 161 | 178 | 8.2 | 0.32 |
| VH:CD ratio (μm/μm) | 2.00 | 1.91 | 2.08 | 0.177 | 0.79 |
| Villus width (μm) | 118 | 112 | 120 | 5.5 | 0.57 |
| **Ileum** |  |  |  |  |  |
| Villus height (μm) | 233 | 223 | 219 | 23.3 | 0.91 |
| Crypt depth (μm) | 176 | 176 | 179 | 9.8 | 0.96 |
| VH:CD ratio (μm/μm) | 1.63 | 1.48 | 1.41 | 0.143 | 0.55 |
| Villus width (μm) | 127 | 131 | 125 | 5.5 | 0.76 |

^1^VH:CD = villus height (μm)/crypt depth (μm).

Control, liquid starter diet provided as creep feed from day 8 of age to weaning; Glutamine, control supplemented with 10 g of L-glutamine per kg of starter diet; Enzymes, control supplemented with a cocktail of enzymes (lipase, protease and α-amylase)
